# Comparative transcriptome analysis of pigeonpea, *Cajanus cajan* (L.) and one of its wild relatives *Cajanus platycarpus* (Benth.) Maesen

**DOI:** 10.1371/journal.pone.0218731

**Published:** 2019-07-03

**Authors:** Maniraj Rathinam, Pragya Mishra, Madavan Vasudevan, Roli Budhwar, Ajay Mahato, A. Lakshmi Prabha, Nagendra Kumar Singh, Uma Rao, Rohini Sreevathsa

**Affiliations:** 1 ICAR-National Institute for Plant Biotechnology, Pusa Campus, New Delhi, India; 2 Department of Botany, School of Life Sciences, Bharathidasan University, Tiruchirapalli, Tamilnadu, India; 3 Bionivid Technology Private Limited, Near New Horizon College, Kasturi Nagar, Bangalore, India; 4 Division of Nematology, ICAR-Indian Agricultural Research Institute, Pusa, New Delhi, India; National Institute of Plant Genome Research, INDIA

## Abstract

Pigeonpea is a major source of dietary protein to the vegetarian population of the Indian sub-continent. Crop improvement to mitigate biotic and abiotic stresses for realization of its potential yield and bridging yield gap is the need of the hour. Availability of limited genomic resources in the cultivated germplasm, however, is a serious bottleneck towards successful molecular breeding for the development of superior genotypes in pigeonpea. In view of this, improvement of pigeonpea can be attempted through transgenesis or by exploiting genetic resources from its wild relatives. Pigeonpea wild relatives are known to be bestowed with agronomic traits of importance; discovery and deployment of genes from them can provide a lucrative option for crop improvement. Understanding molecular signatures of wild relatives would not only provide information about the mechanism behind desired traits but also enable us to extrapolate the information to cultivated pigeonpea. The present study deals with the characterization of leaf transcriptomes of *Cajanus cajan* and one of its wild relatives, *Cajanus platycarpus*. Illumina sequencing revealed 0.11 million transcripts in both the species with an annotation of 0.09 million (82%) transcripts using BLASTX. Comparative transcriptome analyses on the whole, divulged cues about the wild relative being vigilant and agile. Gene ontology and Mapman analysis depicted higher number of transcripts in the wild relative pertaining to signaling, transcription factors and stress responsive genes. Further, networking between the differentially expressed MapMan bins demonstrated conspicuous interactions between different bins through 535 nodes (512 Genes and 23 Pathways) and 1857 edges. The authenticity of RNA-seq analysis was confirmed by qRT-PCR. The information emanating from this study can provide valuable information and resource for future translational research including genome editing to alleviate varied stresses. Further, this learning can be a platform for in-depth investigations to decipher molecular mechanisms for mitigation of various stresses in the wild relative.

## Introduction

Escalating global food demand and the potential impact of climate change have created an increased need for effectual crop improvement programmes. Management of resilience to an array of biotic and abiotic stresses and improvement of productivity through appropriate utilization of available genetic resources requires attention [1–3]. Considering this, development of crops with increased tolerance/resistance to environmental stresses can be a promising option for improvement of food security and agricultural sustainability.

Pigeonpea [*Cajanus cajan* (L.) Millspaugh], also known as red gram, is the sixth most important grain legume crop grown in the semi-arid tropics of Asia, Africa and the Caribbean [4]; India being the largest producer and consumer globally. Productivity of pigeonpea has stagnated over the past years [5] due to a range of vagaries under the scenario of changing climatic conditions. Hence, the major challenge for pigeonpea improvement is not only increasing productivity but also stress mitigation.

Wild relatives, the ancestors of crop plants have been exposed to natural challenges compared to their cultivated counterparts. Continuous exposure to severe climatic conditions has helped them evolve at the genetic level, making them not only more genetically diverse but also more resilient towards the adverse impacts of climate change and incidence of pests and diseases [6]. It is known that pigeonpea was domesticated >3,500 years ago from its wild progenitor, *Cajanus cajanifolius* Maesen in peninsular India [7]. The genus *Cajanus* is composed of 34 species [8], amongst which *C*. *cajan* is the only cultivated member, while the wild relatives are assigned to secondary or tertiary gene pools [9]. Studies have demonstrated that despite possessing unexploited resources that help mitigate a range of stresses, wild relatives of pigeonpea have remained under-utilized due to linkage drag and cross-incompatibility with the cultivated species [10–13].

*Cajanus platycarpus* from the tertiary gene pool is one of the wild relatives of *C*. *cajan* found growing along the hedges with slender and climbing plant type. The leaves and pods are extremely pubescent and produce rectangular dark brown seeds on maturity. *C*. *platycarpus* has the same chromosome number as that of cultivated pigeonpea (*2n* = 22) [14] and is bestowed with various agronomic traits of importance [15–18]. Several characters of this wild relative both at biochemical and morphological levels have been deciphered by scientists worldwide [6, 13, 19, 20]. Despite being laden with resources, cross incompatibility issues led to it not being exploited by conventional techniques [11, 21]. It is therefore evident that through plant domestication, pigeonpea might have lost many of these useful traits present in wild relatives [7] as humans used them to develop plants to suite morphological or physiological traits such as overall yield and edibility [22,23]. This strategically delineates the increased need for not only securing the resources contained in the wild relatives but also exploit its varied applicability in crop improvement programmes. The major focus of our group has been in the area of crop improvement of pigeonpea for pod borer resistance [24, 25] and the broader perspective being deciphering the molecular mechanism of pod borer resistance in *C*. *platycarpus*. In view of this, the major aim of the study has been the characterization of baseline transcriptomes of the cultivated and wild pigeonpea as it can provide fascinating insights on the basic differences in their molecular signatures. Such comparative baseline transcriptomes have been developed in numerous important crops as a step towards broadening the genetic base and better molecular understanding [26–28].

Corroboratory efforts have been made in various crops for extrapolation of the information obtained through molecular characterization of crop wild relatives for revelation of traits endowed to them both under stressed and non-stressed conditions [6, 11, 29–31]. However, in depth depiction to support the use of pigeonpea wild relative towards broadening genetic diversity in the crop has not been embarked upon thus far. Hence, this study, the first of its kind, primarily highlights on the characterization of the baseline transcriptomal differences in *C*. *cajan vis a vis C*. *platycarpus*.

## Materials and methods

### Plant material

Two species of pigeonpea, *C*. *cajan* (cultivated pigeonpea cv. TTB7, a high yielding medium duration variety) procured from UAS, GKVK, Bangalore, India and *C*. *platycarpus* (ICPW 068, a wild relative of pigeonpea) procured from ICRISAT, Hyderabad, India were used in the present study. Seeds of both the species were sown in plastic pots (14 inch diameter and 60 inch height) and maintained under greenhouse conditions. In order to obtain enough plant material for RNA isolation, at least two plants were maintained per pot. Fully expanded and healthy leaves from 3^rd^ or 4^th^ positions were collected from 45 days old plants. Samples were collected from six different plants separately and made into two individual pools; two such pooled samples were considered as replicates. The samples were frozen in liquid nitrogen and stored at -80°C until use.

### RNA extraction, cDNA synthesis, library preparation and sequencing

Total RNA was extracted from *C*. *cajan* and *C*. *platycarpus* leaf samples using Spectrum Plant Total RNA kit (Sigma) following manufacturer’s instructions. RNA samples (5μg) were later treated with DNase to remove the residual genomic DNA and integrity was checked on 1% formaldehyde agarose gel. Total RNA quality control was performed using Agilent 2100 Bioanalyzer (Agilent Technologies, SantaClara, USA) and samples with an RNA integrity number (RIN) of 8.0 were used for mRNA purification. mRNA was purified from 1 μg of intact total RNA using oligodT beads (TruSeq RNA Sample Preparation Kit, Illumina). The purified mRNA was fragmented at an elevated temperature (90 ^0^C) in the presence of divalent cations and reverse transcribed with Superscript II Reverse Transcriptase (Invitrogen Life Technologies) by priming with random hexamers. Second strand cDNA was synthesized in the presence of DNA polymerase I and RNaseH. The cDNA was further cleaned using Agencourt Ampure XP SPRI beads (Beckman Coulter) and Illumina adapters were ligated after end repair and addition of an ‘A’ base followed by SPRI clean-up. The resultant cDNA library was amplified using PCR for enrichment of adapter ligated fragments, quantified using a Nanodrop spectrophotometer (Thermo Scientific) and validated for quality with a Bioanalyzer (Agilent Technologies). The cDNA library was sequenced using Illumina Hi-Seq 2500 platform with 100 bp read length obtained in paired end module. Paired end FASTQ files were subjected to standard quality control with Phred Score >20 using NGSQC Tool Kit [32] to obtain high quality (HQ) filtered reads.

### De Novo transcriptome assembly and analysis

All the HQ filtered paired end libraries were subjected to *de novo* transcriptome assembly. For this study, we chose *de novo* bruijn graph-based Trinity Assembler [33] with criteria, a) default K-mer, b) less memory foot print, c) optimized for Illumina paired end data, d) reproducibility and e) configurable for all computing capacities. The assembled transcripts with length >200 bp were then clustered using CD-HIT-EST tool to obtain non-redundant transcripts set [34]. Clustered transcripts of >200 bp length were considered as secondary assembly and taken further for annotation and expression profiling.

### Annotation and quantification of the transcriptome

Annotation of the unique transcripts (>200 bp) was performed using BLASTX homology search against NCBI non-redundant (nr) protein database (Protein BLAST: http://blast.ncbi.nlm.nih.gov/Blast.cgi?PAGE=Protein). BLAST hits with e-value cutoff ≤ 1e^-14^ and query coverage of >80% were considered as annotated homologous proteins and AWK script was used for filtering reciprocal best hits. BLAST hits were processed to retrieve associated Gene Ontology (GO) terms describing biological processes, molecular functions, and cellular components. Expression levels of all the transcripts in the individual libraries in replicates were assessed by mapping high quality (HQ) filtered reads using BOWTIE2 [35]. Mapped reads were further normalized using Fragments Per Kilo base Per Millions (FPKM) method.

### Functional annotation of specific and differentially expressed transcripts

Transcripts annotated in both the *Cajanus* spp. were individually plotted as pie-donut charts for five major GO terms and pathways by using an online server (Highcharts: https://www.highcharts.com) followed by GO categorization using another online server, Wego [36]. For MapMan [37] analysis, transcripts expressed in both the species were annotated with TAIR database (Arabidopsis homologs). BIN level information and gene identifiers were also derived from the same database.

Differential gene expression analysis of the expressed transcripts was performed using DESeq [38] software based on R programming environment. Transcripts that were ≥2 fold up or down-regulated with a p-Value (Derived by Student t-test) of ≤0.05 indicative of FDR were considered as differentially expressed. Unsupervised hierarchical clustering of up and down-regulated genes was performed using Cluster 3.0 software [39] by applying Pearson uncentered algorithm with average linkage rule. Further, clusters of transcripts were visualized using Java Tree View software [40] to identify the pattern of up and down-regulated transcripts. Isotig analyses of annotated transcripts were carried out to understand redundancy in each of the libraries. Biological analysis of differentially expressed genes was performed based on GO annotations obtained from EBI-GOA database [41] and KEGG Pathway database [42].

### Network modeling of differentially expressed genes

Enriched biological categories along with differentially expressed genes were used as input for Bridge Island Software (Bionivid Technology Pvt Ltd, Bangalore, India) for identifying key edges that connect genes and biological categories. Statistical scores from differential expression and biological analyses were used as attributes to visualize the network. Output of Bridge Island Software was used as input to CytoScape V 2.8 [43]. The nodes were colored based on the Log 2 fold change values of genes representing induction (red) and repression (green) between the two *Cajanus* spp and pathway clusters.

### Validation of the transcriptome data by qRT PCR

About 2 μg total RNA was used for cDNA synthesis by Superscript Vilo cDNA synthesis kit (Invitrogen). The diluted cDNA was used as a template in qRT PCR and amplified with gene specific primers (Table A in S1 File) using SYBR Green PCR master mix on AriaMx Real-Time PCR system (Agilent USA) according to the manufacturer’s instructions. Expression of *IF4α* gene in each sample was used for normalization. RT PCR conditions were set as: initial denaturation at 95°C for 5 min, followed by 40 cycles each of 95°C for 10 sec, 60°C for 15s and 72°C for 15s. qRT-PCR was performed in two independent biological replicates with three technical replicates along with no template control. For analysis, *C*. *platycarpus* was considered as the test and *C*. *cajan* as control. The data was first normalized by subtracting internal reference gene from test and control samples and fold change was calculated [44].

## Results

**Fig 1:**
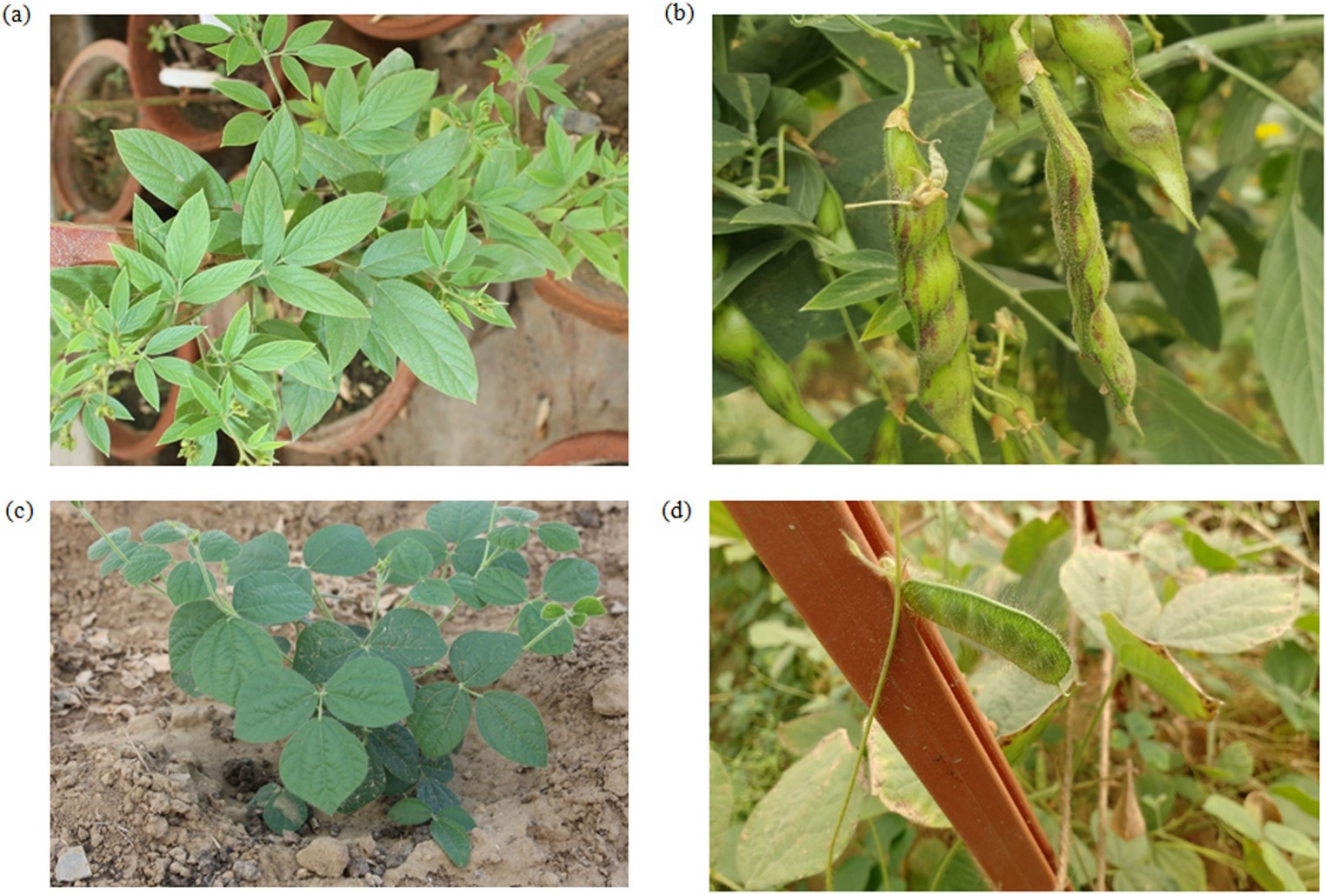
(a—b) morphology of cultivated pigeonpea (*C*. *cajan*) plant and their pods respectively. (c—d) morphology of the wild relative of pigeonpea (*C*. *platycarpus*) plant and their pods respectively.

*C*. *platycarpus* is an incompatible wild species from the tertiary gene pool of pigeonpea and morphologically distinct from *C*. *cajan*. While the domesticated pigeonpea portrays tall bushy plants that bear flowers at the end of the branches (Fig 1A and 1B), *C*. *platycarpus* are climbing plants that are slender and pubescent (Fig 1C and 1D).

### Transcriptome sequencing and assembly

**Fig 2:**
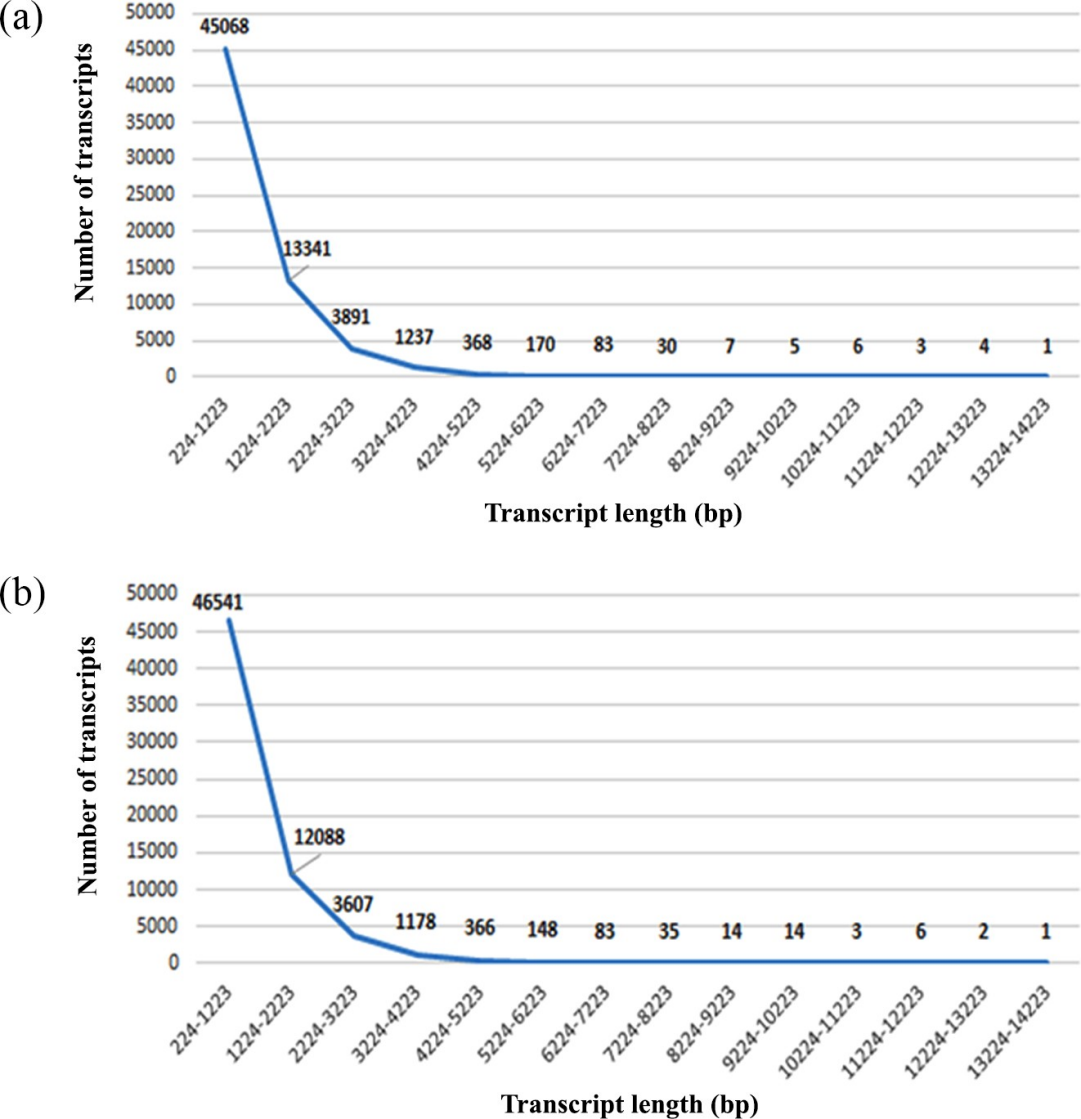
**Transcript length distribution analysis of** (a) *C*. *cajan* and (b) *C*. *platycarpus* libraries.

Subsequent to RNA sequencing, reads having ≥70% of the bases with a quality score ≥Q20 were selected using NGS QC Toolkit. An average of 16 million high quality reads per cDNA library with a QScore of ≥Q20 was obtained of which 97% of reads were of high quality (Table B in S1 File). We also observed that the cDNA libraries had an average of 46% GC content in both the transcriptomes (Table B in S1 File). Transcriptome assembly resulted in an average of 64,000 transcripts in both *C*. *cajan* and *C*. *platycarpus* transcriptomes individually. The total transcriptome accounted for an average of 65 Mb with a minimum transcript length of 200 bp and maximum of 13.5 kb with a N50 value of 1.4 kb, indicating an optimized unfragmented transcriptome assembly. In order to obtain a non-redundant transcriptome for both the species, transcripts from individual assemblies were subjected to CD HIT EST clustering analysis. Further, upon combining the two non-redundant assemblies, 114781 transcripts accounting for 113 megabases were obtained without any change in the minimum and maximum transcript length and N50 value (Table C in S1 File). The authenticity of the combined transcript assembly was validated by aligning the reads from the replicate cDNA libraries of both the species to all 114781 transcripts using Bowtie 2 (Table B in S1 File). Transcript length distribution analysis of *C*. *cajan* and *C*. *platycarpus* libraries showed ~70% of the transcripts having an average of 1 Kb length (Fig 2A and 2B).

### Functional annotation of the transcriptome

**Fig 3:**
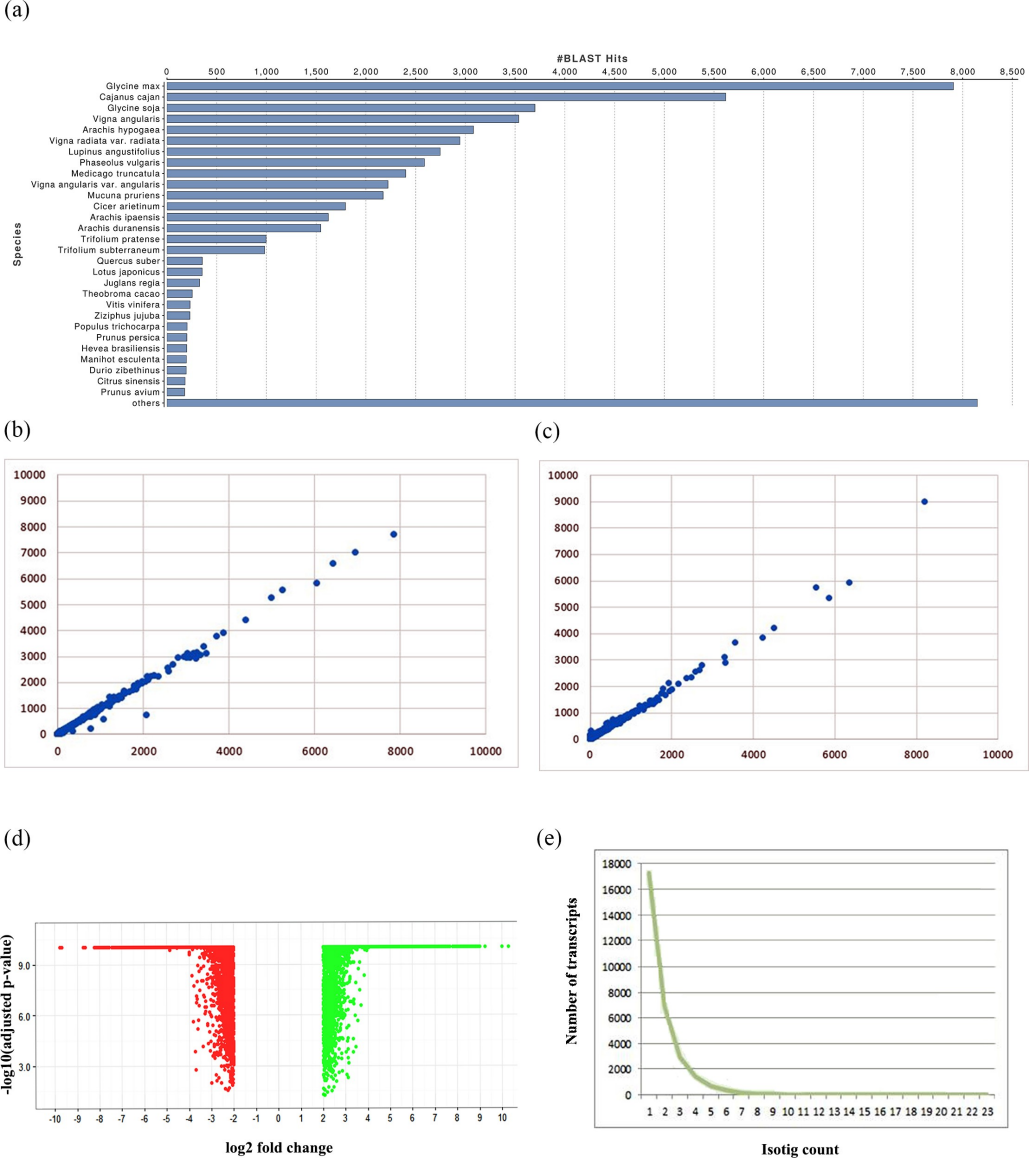
(a) Species distribution of transcripts; Correlation covariance analysis depicting correlation between the biological replicates of (b) *C*. *cajan* and (c) *C*. *platycarpus*; (d) Uniform distribution of up- and down- regulated transcripts in *C*. *cajan* in comparison to *C*. *platycarpus* as illustrated by the volcano plot; (e) Distribution of Isotigs in the combined transcriptomes.

According to BLASTX scores using an e value cutoff of 1e^-14^ and minimum query coverage of 80%, 94136 (82%) transcripts were annotated (Table A in S1 Data). These annotated ESTs were found to match with multiple plant species (Fig 3A) and maximum similarity was obtained with *Glycine max* followed by *C*. *cajan*.

### Transcript expression profiling and comparative transcriptome analysis

Read counts for all the 114781 transcripts obtained from the validated transcriptome were provided as input to DESEQ pipeline for normalization and expression profiling and further classified as up-regulated, down-regulated, *C*. *cajan-*specific and *C*. *platycarpus-*specific. Expression was detected in 68980 (60%) transcripts with <10 Read count out of the total 114781 transcripts. We also observed 13203 (11.5%) transcripts to be *C*. *platycarpus-*specific and 11402 (10%) to be *C*. *cajan*-specific (Table A in S1 Data). Differential expression analysis with pValue <0.05 and a fold change cutoff of ≥2 as up-regulated and ≤-2 as down-regulated resulted in the identification of 9151 (8%) transcripts to be up and 8580 (7.52%) transcripts down-regulated (Table B in S1 Data). Correlation covariance analysis of replicate samples in *C*. *cajan* and *C*. *platycarpus* showed an R^2^ value of 0.956 for *C*. *cajan* replicate samples and 0.991 for *C*. *platycarpus* replicate samples (Fig 3B and 3C), indicating a very high degree of biological replicate reproducibility. Further, volcano plot depicted uniform distribution of up and down-regulated transcripts in *C*. *cajan* compared to *C*. *platycarpus* indicating trait specific gene regulation (Fig 3D). Furthermore, Isotig analysis revealed >90% of the genes expressed were represented by only 1 transcript indicating a very high degree of integrity (Fig 3E). All clean reads were deposited in the NCBI Short Read Archive (SRA) database and can be accessed with accession numbers—SRR6785591, SRR6785590, SRR6785593, SRR6785592.

### Functional enrichment analysis

**Fig 4:**
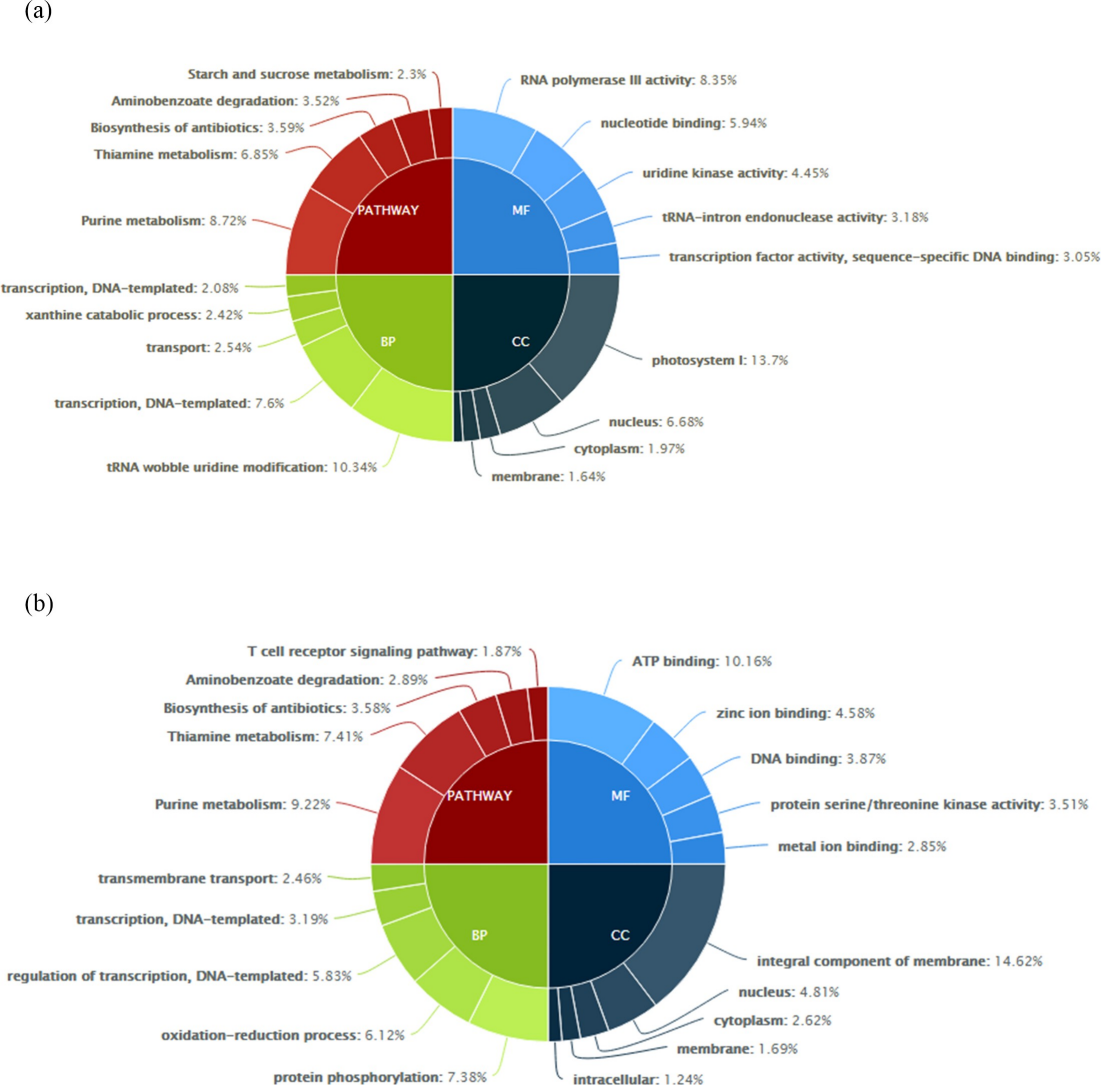
**Classification of top five Gene Ontology (GO) categories of annotated transcripts in** (a) *C*. *cajan* and (b) *C*. *platycarpus*.

Transcriptomal variation between the two species was analyzed by GO categorization of all the transcripts into four major categories *viz*., cellular components, biological process, molecular function and pathways (Fig 4A and 4B). Though major differences were not observed in the category cellular components, genes related to photosystem I were seen to be major in *C*. *cajan* (Fig 4A) whereas, genes related to integral components of membrane were prominent in *C*. *platycarpus* (Fig 4B). Further, with respect to molecular function, genes related to transcriptional activity like RNA polymerase III, nucleotide binding, uridine kinase activity, tRNA-intron endonuclease activity and transcription factors were shown to be more in *C*. *cajan*. An entirely different situation was however observed in *C*. *platycarpus* as the genes concerned with ATP binding, zinc ion binding, DNA binding, serine/threonine kinases and metal ion binding were seen to be enriched.

**Fig 5:**
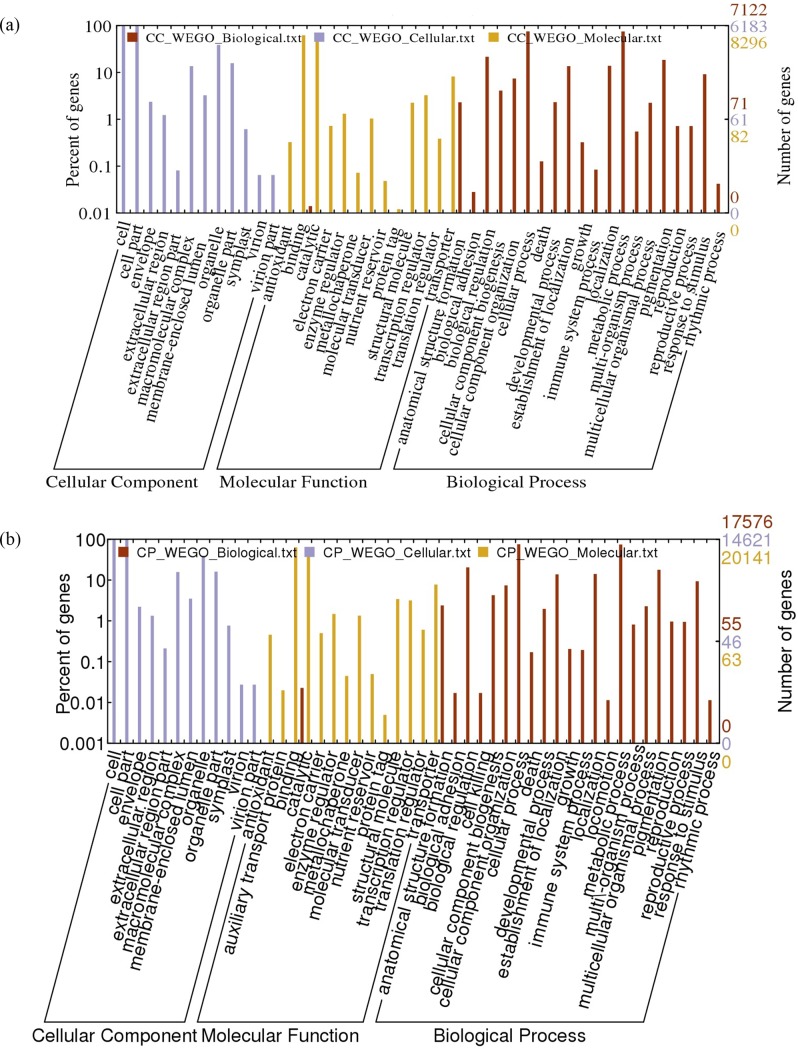
**Gene Ontology (GO) classification of annotated transcripts in** (a) *C*. *cajan* and (b) *C*. *platycarpus*.

In the GO category of pathways, majority of genes belonging to purine and thiamine metabolism, biosynthesis of antibiotics and aminobenzoate degradation were seen to be expressed in both the systems. Interestingly, genes related to carbohydrate metabolism were dominating in *C*. *cajan* whereas, T-cell receptor signaling pathway genes were more in *C*. *platycarpus* (Fig 4A and 4B). Further, profound variation between the two systems was observed in the GO category of biological processes. While genes related to transcription and related processes like tRNA wobble uridine modification and transcription were predominant in the cultivated pigeonpea, protein phosphorylation, oxidation–reduction process and transcription were seen to be more in *C*. *platycarpus* (Fig 4A and 4B). The number of transcripts pertaining to each of the GO categories was also seen to be differing between the two systems as evidenced in Fig 5A and 5B.

### Functional characterization by MapMan analysis

**Fig 6:**
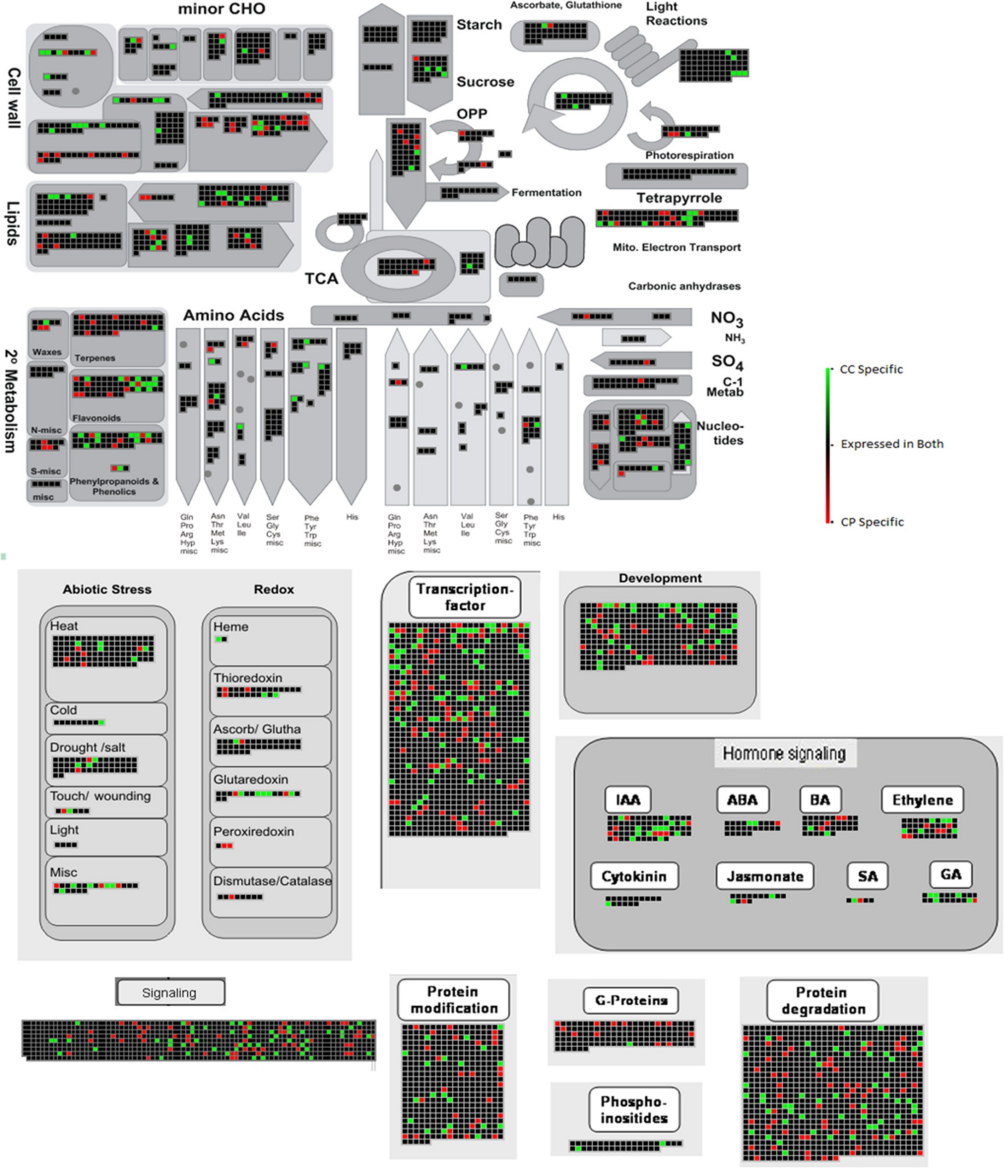
MapMan analysis depicting gene expression in functional categories associated with different pathways in both the *Cajanus* species.

For comprehensive assessment of variations/similarities in the transcriptomes of both the species, transcripts were mapped by MapMan tool and were separated by bins based on their functional ontology (Fig 6). Fascinatingly, significant variation was observed between the two systems with respect to genes related to important functions like those involved in different aspects of signaling *viz*., G proteins, kinases etc; genes related to secondary metabolism which are pertinent in response of plants to various environmental stimuli; genes related to translation of the perceived stimuli in terms of DNA binding. An in-depth analysis of genes expressed in these categories that actually demarcate the two species was considered as relevant and major emphasis was therefore given on the following bins.

### Transcripts related to transcription factors

MapMan analysis depicted that totally 1060 transcripts were mapped from both the species; 514 and 379 transcripts being specific to *C*. *platycarpus* and *C*. *cajan* respectively. Though major differences in the kind of transcripts were not observed between the two species, heat shock transcription factors were seen to be expressed more in number in *C*. *platycarpus* (S2 Data). About 125 transcripts were differentially expressed in both species and more than 78 transcripts among those were up-regulated in *C*. *platycarpus*. Further, WRKY and MYB transcription factors (TFs) were seen to be expressed in more numbers compared to other TFs. Comparatively, WRKY transcription factors showed higher expression in *C*. *platycarpus* whereas more number of MYB TFs displayed higher expression in *C*. *cajan*. However, highest expression level of both the transcription factors was seen in *C*. *platycarpus*. In contrast to MYB, TFs belonging to homeobox-leucine zipper were found to be expressing more in *C*. *cajan* (Table 1; S3 Data). Further, analysis depicted that bZIP protein, BEL1-like homeodomain protein 6, calmodulin-binding transcription activator 1-like isoform X2, E2F transcription factor-like E2FE isoform X2, ethylene insensitive 3-like 1 protein and GATA transcription factor 7-like TFs were present in higher levels in *C*. *platycarpus*. On the other hand, heat stress transcription factors, MADS-box protein SOC1-like isoform X1, protein LHY isoform X3, protein REVEILLE 7 and scarecrow-like protein 14 isoform X1 transcription factors were seen to be expressing at higher levels in *C*. *cajan*.

**Table 1:** Representative transcripts of transcription factors differentially expressed in *C*. *platycarpus* compared to *C*. *cajan*.

Transcript ID	log2 fold change	Transcript description
CP_TR48453|c0_g1_i1_len = 1796	7.0	PREDICTED: ETHYLENE INSENSITIVE 3-like 1 protein
CP_TR2864|c0_g1_i1_len = 1587	5.2	PREDICTED: trihelix transcription factor GTL2-like
CP_TR23396|c0_g1_i1_len = 1249	5.0	PREDICTED: GATA transcription factor 7-like
CP_TR2657|c0_g2_i3_len = 1176	4.9	PREDICTED: myb-related protein Myb4-like
CP_TR46143|c0_g1_i2_len = 872	4.9	WRKY65
CP_TR8901|c0_g1_i1_len = 1585	4.9	PREDICTED: probable WRKY transcription factor 41
CP_TR24329|c0_g1_i1_len = 1886	4.9	PREDICTED: probable WRKY transcription factor 3
CP_TR38258|c0_g2_i1_len = 985	4.5	hypothetical protein PHAVU_009G004800g
CP_TR38258|c0_g1_i1_len = 726	4.1	PREDICTED: BEL1-like homeodomain protein 6
CP_TR29370|c0_g3_i1_len = 3564	4.0	PREDICTED: calmodulin-binding transcription activator 1-like isoform X2
CP_TR27324|c0_g1_i2_len = 1359	3.4	PREDICTED: E2F transcription factor-like E2FE isoform X2
CP_TR15447|c0_g1_i2_len = 1931	3.0	bZIP protein
CP_TR32168|c4_g1_i4_len = 2950	2.7	PREDICTED: homeobox-leucine zipper protein ATHB-8-like
CP_TR10018|c0_g2_i1_len = 968	2.4	PREDICTED: homeobox-leucine zipper protein HDG11-like
CP_TR5066|c0_g1_i1_len = 400	2.0	PREDICTED: calmodulin-binding transcription activator 2-like
CC_TR2425|c0_g1_i1_len = 1079	-2.1	PREDICTED: transcription factor MYB34
CC_TR30934|c0_g1_i4_len = 1348	-2.2	PREDICTED: transcription factor TGA6 isoform X1
CC_TR19500|c0_g1_i1_len = 1535	-2.6	PREDICTED: transcription factor TGA2-like isoform X1
CC_TR27394|c1_g2_i3_len = 1342	-2.7	PREDICTED: myb family transcription factor APL-like
CC_TR4919|c0_g1_i1_len = 2458	-2.9	PREDICTED: scarecrow-like protein 13
CC_TR9284|c0_g1_i1_len = 657	-3.0	hypothetical protein PHAVU_006G182000g
CC_TR22466|c0_g1_i2_len = 591	-3.1	hypothetical protein PHAVU_001G091100g
CC_TR31096|c3_g1_i9_len = 2583	-3.9	PREDICTED: scarecrow-like protein 14 isoform X1
CC_TR13358|c1_g1_i1_len = 795	-4.0	PREDICTED: homeobox-leucine zipper protein ATHB-12-like
CC_TR30641|c0_g1_i4_len = 3236	-4.2	PREDICTED: homeobox-leucine zipper protein ATHB-15
CC_TR25327|c0_g1_i2_len = 1720	-4.7	hypothetical protein PHAVU_005G100700g
CC_TR44167|c0_g1_i1_len = 638	-5.0	PREDICTED: protein LHY isoform X3
CP_TR10174|c0_g2_i1_len = 1176	-5.3	heat stress transcription factor A-6b-like
CP_TR20195|c1_g1_i1_len = 476	-5.8	PREDICTED: protein REVEILLE 7
CC_TR52932|c0_g1_i1_len = 833	-6.0	PREDICTED: MADS-box protein SOC1-like isoform X1

### Transcripts related to signaling and protein modification

With respect to various transcripts belonging to signaling and G- proteins, a total of 6076 transcripts were expressed in both the species. Among them, 977 and 701 transcripts were seen to be specific to *C*. *platycarpus* and *C*. *cajan* respectively. Transcripts belonging to G-proteins, calcium signaling and receptor kinases were specifically found to be dominating in *C*. *platycarpus* (S2 Data). It was observed that 653 transcripts were differentially expressed with 410 transcripts being up-regulated and 243 down-regulated in *C*. *platycarpus* compared to *C*. *cajan*. With respect to the differentially expressed transcripts, those that belonged to calcium mediated signaling and the family of receptor like kinases (RLKs) were seen to be abundant. Among the transcripts pertaining to calcium mediated signaling, calcium-transporting ATPase, calcium-dependent protein kinase, calcium-binding protein, calmodulin-like protein, and calmodulin-binding protein/transcription activators were displayed in higher levels in *C*. *platycarpus*. Nevertheless, calcineurin B-like protein and CBL-interacting serine/threonine-protein kinases were predominantly expressed in *C*. *cajan* (Table 2; S3 Data**).**

**Table 2:** Representative transcripts related to signaling differentially expressed in *C*. *platycarpus* compared to *C*. *cajan*.

Transcript id	Log2 fold change	Transcript description
**Protein kinases, phosphatase and G-proteins**		
CP_TR32556|c0_g1_i5_len = 2652	6.9	G-type lectin S-receptor-like serine/threonine-protein kinase
CP_TR29770|c0_g4_i1_len = 2696	5.3	PREDICTED: putative receptor protein kinase ZmPK1
CP_TR3510|c0_g1_i1_len = 2199	5.0	PREDICTED: probable receptor-like protein kinase At1g67000
CP_TR30988|c0_g3_i2_len = 3286	4.9	PREDICTED: extra-large guanine nucleotide-binding protein 1-like
CP_TR22760|c0_g2_i2_len = 2065	4.9	PREDICTED: wall-associated receptor kinase-like 14
CP_TR21388|c0_g1_i1_len = 1475	4.5	PREDICTED: probable protein phosphatase 2C 78 isoform X1
CP_TR45053|c0_g1_i1_len = 2253	4.3	PREDICTED: L-type lectin-domain containing receptor kinase VII.1
CP_TR30933|c0_g1_i4_len = 3258	4.3	PREDICTED: receptor protein kinase TMK1-like
CP_TR29347|c0_g1_i1_len = 3427	4.3	PREDICTED: LRR receptor-like serine/threonine-protein kinase HSL2
CP_TR32141|c0_g1_i4_len = 2751	4.1	PREDICTED: EVI5-like protein isoform X4
CP_TR443|c0_g2_i1_len = 1474	3.9	PREDICTED: lysM domain receptor-like kinase 3
CP_TR30412|c0_g2_i1_len = 960	3.8	PREDICTED: proline-rich receptor-like protein kinase PERK9
CP_TR29703|c0_g1_i1_len = 3630	3.5	PREDICTED: leucine-rich repeat receptor-like tyrosine-protein kinase PXC3
CP_TR2871|c0_g1_i1_len = 1453	3.3	PREDICTED: rop guanine nucleotide exchange factor 5-like isoform X1
CC_TR30364|c0_g1_i4_len = 1332	-3.1	PREDICTED: TBC1 domain family member 22B isoform X1
CC_TR1558|c0_g2_i1_len = 957	-3.2	PREDICTED: GTP-binding protein SAR1A
CP_TR37415|c0_g1_i1_len = 1865	-4.3	PREDICTED: L-type lectin-domain containing receptor kinase VIII.2-like
CC_TR30321|c0_g1_i4_len = 1904	-4.6	PREDICTED: probable protein phosphatase 2C 40
CC_TR30662|c1_g2_i3_len = 3242	-7.9	PREDICTED: cysteine-rich receptor-like protein kinase 25 isoform X1
**Calcium dependent signaling**		
CC_TR23765|c0_g2_i1_len = 434	4.7	PREDICTED: calcium-transporting ATPase 2, plasma membrane-type-like
CP_TR22900|c0_g1_i1_len = 1333	3.8	PREDICTED: calcium-dependent protein kinase 2
CP_TR14743|c0_g1_i1_len = 2533	3.4	PREDICTED: calmodulin-binding protein 60 E-like isoform X1
CC_TR29574|c0_g1_i6_len = 590	-3.2	PREDICTED: calcineurin B-like protein 10 isoform X3
CC_TR47011|c0_g1_i1_len = 1177	-6.8	PREDICTED: CBL-interacting serine/threonine-protein kinase 10-like

Leucine-rich repeat receptor-like protein kinases (LLR-RLKs) were seen to be abundantly expressed in both the species compared to other RLKs. Distinctively, LLR-RLKs were predominantly expressed in *C*. *platycarpus* along with proline-rich and LysM domain RLKs. Further, probable/putative receptor like kinases and threonine-protein kinases were specifically up-regulated in *C*. *platycarpus* whereas, G-type lectin-RLKs (GsRLKs) showed higher level of expression in *C*. *cajan* (Table 2; S3 Data).

Protein kinases and phosphatases are important regulators of proteins in biological systems. Based on differential gene expression analysis, mitogen-activated protein kinase, casein kinase, receptor protein kinase TMK1, wall-associated receptor kinase, and putative receptor protein kinase ZmPK1 were seen to be specifically up regulated in *C*. *platycarpus*. However, L-type lectin-domain containing receptor kinase was equally expressed in both the species. Different isoforms of protein phosphatases were also found to be up-regulated in both the species.

The study illustrated that 48 transcripts that belonged to G-proteins were differentially expressed in both species, out of which, 27 transcripts were found to be up-regulated in the wild relative. Particularly, the up-regulated transcripts included, extra-large guanine nucleotide-binding protein 1-like, EVI5-like protein isoform X4, ras-related protein Rab11A-like and rop guanine nucleotide exchange factor 5-like isoform X1. In the domesticated pigeonpea, transcripts belonging to 22B isoform X1, a member of TBC1 domain family proteins and GTP-binding protein SAR1A were seen to be up-regulated (Table 2; S3 Data).

### Secondary metabolism

According to MapMan analysis, the bin pertaining to secondary metabolism consisted of 803 transcripts that expressed in both the species; *C*. *platycarpus* and *C*. *cajan* specific transcripts being 160 and 169 respectively. However, no major difference was seen in the types of transcripts between both the species (S2 Data). It was observed that 147 transcripts were differentially expressed in *C*. *platycarpus* compared to *C*. *cajan* wherein, 62 transcripts were up-regulated and others were down-regulated. Among the differentially expressed transcripts, phenylpropanoid and flavonoid biosynthesis pathway genes were predominantly expressed in both species (Table 3; S3 Data). Interestingly, transcript annotated as 4-hydroxyphenylpyruvate dioxygenase displayed higher level of expression in *C*. *platycarpus* (Table 3; S3 Data) indicating improved synthesis of tocopherols in this species as a putative regulator of reactive oxygen species.

**Table 3:** Representative transcripts related to secondary metabolite synthesis pathways differentially expressed in *C*.*platycarpus* compared to *C*. *cajan*.

Transcript ID	Log2 fold change	Transcript description
**Phenylprophanoids metabolisam**		
CP_TR441|c0_g1_i1_len = 1967	4.1	PREDICTED: 4-coumarate--CoA ligase-like 9
CP_TR16870|c0_g1_i1_len = 1265	3.6	PREDICTED: cinnamoyl-CoA reductase 2-like
CP_TR21053|c0_g3_i1_len = 1054	2.8	PREDICTED: probable caffeoyl-CoA O-methyltransferase At4g26220
CP_TR15201|c0_g1_i1_len = 1560	2.3	PREDICTED: phenylalanine ammonia-lyase class 3 isoform X1
CC_TR79|c0_g1_i1_len = 1250	-2.4	PREDICTED: phenylalanine ammonia-lyase 1
CP_TR8874|c0_g2_i1_len = 685	-3.7	PREDICTED: shikimate O-hydroxycinnamoyltransferase isoform X3
CC_TR18917|c0_g1_i1_len = 1236	-4.1	PREDICTED: cinnamoyl-CoA reductase 1-like
CC_TR18296|c0_g1_i1_len = 1729	-5.1	PREDICTED: probable cinnamyl alcohol dehydrogenase
CC_TR50583|c0_g1_i1_len = 1614	-5.7	PREDICTED: shikimate O-hydroxycinnamoyltransferase isoform X2
CC_TR21336|c0_g2_i1_len = 2198	-6.8	PREDICTED: 4-coumarate--CoA ligase 1
**Flavonids metabolisam**		
CP_TR7759|c0_g2_i1_len = 1325	6.2	PREDICTED: anthocyanidin 3-O-glucosyltransferase 5-like
CP_TR37480|c0_g1_i1_len = 1762	5.2	PREDICTED: flavonoid 3&apos;-monooxygenase-like
CP_TR49870|c0_g1_i1_len = 468	4.4	PREDICTED: chalcone synthase 5
CP_TR26778|c0_g1_i2_len = 795	4.4	flavonol synthase/flavanone 3-hydroxylase
CP_TR24400|c0_g1_i5_len = 1557	3.5	PREDICTED: putative dihydroflavonol-4-reductase
CP_TR12032|c0_g1_i1_len = 1308	3.5	anthocyanidin synthase
CC_TR27457|c0_g1_i1_len = 1684	-3.4	PREDICTED: anthocyanidin 3-O-glucosyltransferase 5-like
CC_TR24066|c0_g2_i1_len = 939	-3.6	PREDICTED: dihydroflavonol-4-reductase-like
CC_TR36517|c0_g1_i1_len = 1031	-4.4	PREDICTED: chalcone--flavonone isomerase-like
CC_TR45250|c0_g1_i1_len = 1623	-4.9	Anthocyanidin 3-O-glucoside 2&apos;&apos;-O-glucosyltransferase-like
CC_TR45038|c0_g1_i1_len = 1222	-5.6	flavonol synthase
CC_TR19556|c0_g1_i2_len = 1527	-5.6	PREDICTED: leucoanthocyanidin dioxygenase
**Terpenoids metabolisam**		
CP_TR4750|c0_g1_i1_len = 2429	2.8	PREDICTED: probable 1-deoxy-D-xylulose-5-phosphate synthase 2, chloroplastic
CP_TR24428|c0_g1_i3_len = 2136	2.1	PREDICTED: 3-hydroxy-3-methylglutaryl-coenzyme A reductase 1-like
CC_TR6946|c0_g1_i1_len = 1805	-3.5	1-deoxy-D-xylulose 5-phosphate reductoisomerase, chloroplastic-like
CC_TR13227|c0_g2_i1_len = 1545	-4.4	PREDICTED: geranylgeranyl pyrophosphate synthase, chloroplastic-like
CC_TR22580|c0_g1_i4_len = 750	-4.6	PREDICTED: beta-amyrin synthase
CC_TR10054|c0_g1_i1_len = 2017	-6.6	glucosyltransferase
CC_TR4266|c0_g1_i1_len = 2042	-10.0	PREDICTED: isoprene synthase, chloroplastic-like
**Tocopherol metabolisam**		
CP_TR10466|c0_g2_i1_len = 1729	4.0	PREDICTED: 4-hydroxyphenylpyruvate dioxygenase
CP_TR22271|c0_g1_i1_len = 1375	3.8	PREDICTED: tocopherol O-methyltransferase, chloroplastic-like

Stress related genes

MapMan bins for abiotic stress, redox and other stresses showed that 2056 transcripts were mapped amongst which 186 and 105 were expressed specific to *C*. *platycarpus* and *C*. *cajan* respectively (S2 Data). Around 210 transcripts were differentially expressed in *C*. *platycarpus* compared to *C*. *cajan* wherein, 115 were up-regulated and the remaining down-regulated (S3 Data). Transcripts from nucleotide binding and leucine rich repeats (NBS-LRR) family were seen to be abundantly expressed in both the species (Table 4) along with PR proteins.

**Table 4:** Representative transcripts belonging to stress response genes differentially expressed in *C*. *platycarpus* compared to *C*. *cajan*.

Transcript id	Log2 fold change	Transcript description
**Biotic stress**		
CP_TR32736|c3_g3_i3_len = 3757	6.8	disease resistance protein (TIR-NBS-LRR class)
CP_TR39176|c0_g1_i1_len = 1035	4.9	PR-5b protein precursor
CP_TR37702|c0_g1_i1_len = 1077	4.6	PREDICTED: acidic endochitinase-like
CP_TR30626|c0_g4_i1_len = 3795	4.4	PREDICTED: TMV resistance protein N-like
CP_TR31255|c0_g1_i4_len = 4069	4.1	LRR and NB-ARC domain disease resistance protein
CP_TR30484|c0_g1_i4_len = 3219	3.9	PREDICTED: disease resistance protein RPM1-like
CP_TR28412|c0_g1_i2_len = 1372	3.4	PREDICTED: thaumatin-like protein 1
CP_TR14448|c0_g1_i1_len = 1269	2.2	PREDICTED: lipase-like PAD4 isoform X1
CC_TR29841|c0_g1_i8_len = 1949	-3.1	PREDICTED: putative disease resistance protein RGA3
CC_TR31064|c0_g1_i2_len = 4039	-4.3	PREDICTED: putative disease resistance RPP13-like protein 1
CC_TR21614|c0_g1_i1_len = 1308	-4.9	PREDICTED: chitinase-like protein 2
**Abiotic stress**		
CP_TR23836|c0_g1_i2_len = 1334	5.6	PREDICTED: probable methyltransferase PMT19
CP_TR20408|c0_g3_i1_len = 1509	2.5	PREDICTED: ultraviolet-B receptor UVR8-like isoform X2
CC_TR1092|c0_g1_i1_len = 1396	-3.9	PREDICTED: dnaJ protein homolog 1-like
CC_TR50239|c0_g1_i1_len = 3229	-4.1	heat shock protein
CC_TR22001|c0_g2_i1_len = 2595	-6.3	PREDICTED: heat shock protein 83-like

Network analysis

With respect to the genes related to abiotic stress, expression of heat shock proteins and heat stress transcription factors was observed in both the species, except for heat stress transcription factor B-2b which was not up-regulated in *C*. *cajan* (Table 4; S3 Data). Further, heat shock related chaperone, dnaJ protein homologs were seen to be equally expressed in both the species. Besides, common stress related genes such as probable methyltransferase genes were also seen to be predominantly expressing in *C*. *platycarpus* (Table 4; S3 Data).

**Fig 7:**
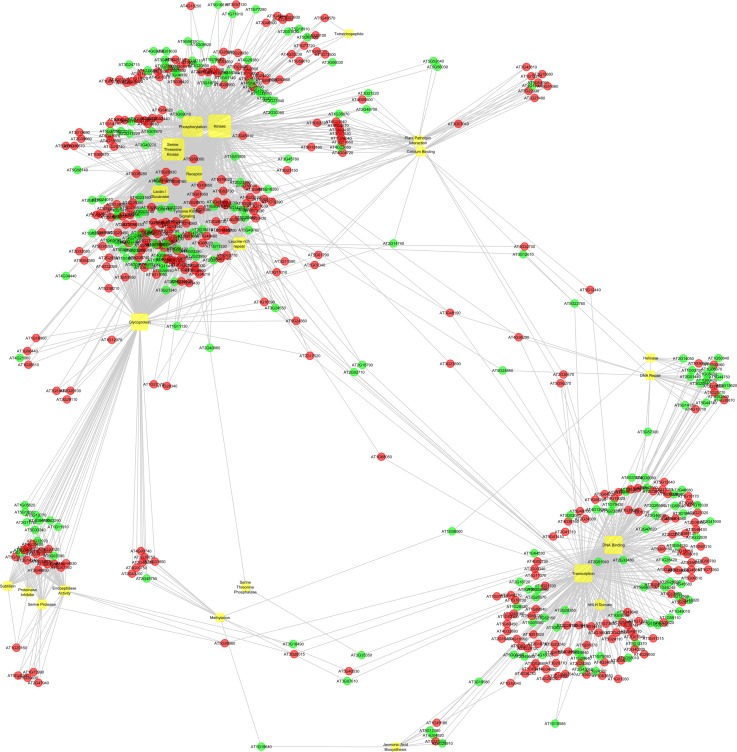
Biological categories-based network analysis depicting connection between the genes mapped in different MapMan bins.

The bins which consisted of significantly varying transcripts between the two species were deciphered further for a better understanding of the scenario in the wild relative. Towards this, a total of 23 biologically important pathways comprising of 512 significantly expressed genes were found and presented in the network consisting of 535 nodes (512 Genes and 23 Pathways) and 1857 edges (S4 Data). It was explicit that the transcripts in the respective bins were strongly interacting (Fig 7) with three distinct clusters evident in the network (Fig 7) showing both up- as well as down-regulation. Interestingly, network analysis demonstrated co-ordination of signal perception and its transduction in the pigeonpea wild relative. Cluster one deciphered the nature of interaction happening in the signaling category and was seen to be a dense cluster with more number of nodes and many of them up-regulated in *C*. *platycarpus*. Specifically, the nodes consisted of biological categories like, glycoprotein, lectin/glucanases, receptors, serine/threonine kinases, tyrosine kinase signaling, lectine-rich repeats, phosphorylation and kinases. Further, a group of genes that displayed higher expression in *C*. *platycarpus* in the same cluster were also shared by two other biological categories, plant pathogen interaction and calcium binding. These two biological categories consisted of some other nodes which were not shared by cluster one but displayed up-regulation in *C*. *platycarpus* (Fig 7). The second cluster consisted mainly of biological processes related to the transduction of signal in terms of transcription factors. Nodes that belonged to DNA binding and bHLH domain that were present in cluster 2 were shown to be up-regulated in the wild relative. A sub cluster present in cluster 2 was shared by biological categories such as helicase and DNA repair displaying down-regulation in *C*. *platycarpus*. Cluster 3 consisted of lesser number of nodes that belonged to biological categories such as subtilisin, proteinase inhibitor, serine protease and endopeptidase activity that are primarily concerned with protein catabolic regulation. Though this cluster had lesser number of nodes, half of the genes were up-regulated in *C*. *platycarpus* while genes shared by endopepdidase activity displayed down-regulation in the wild relative.

### Validation of RNA-seq analysis by qRT-PCR

**Fig 8:**
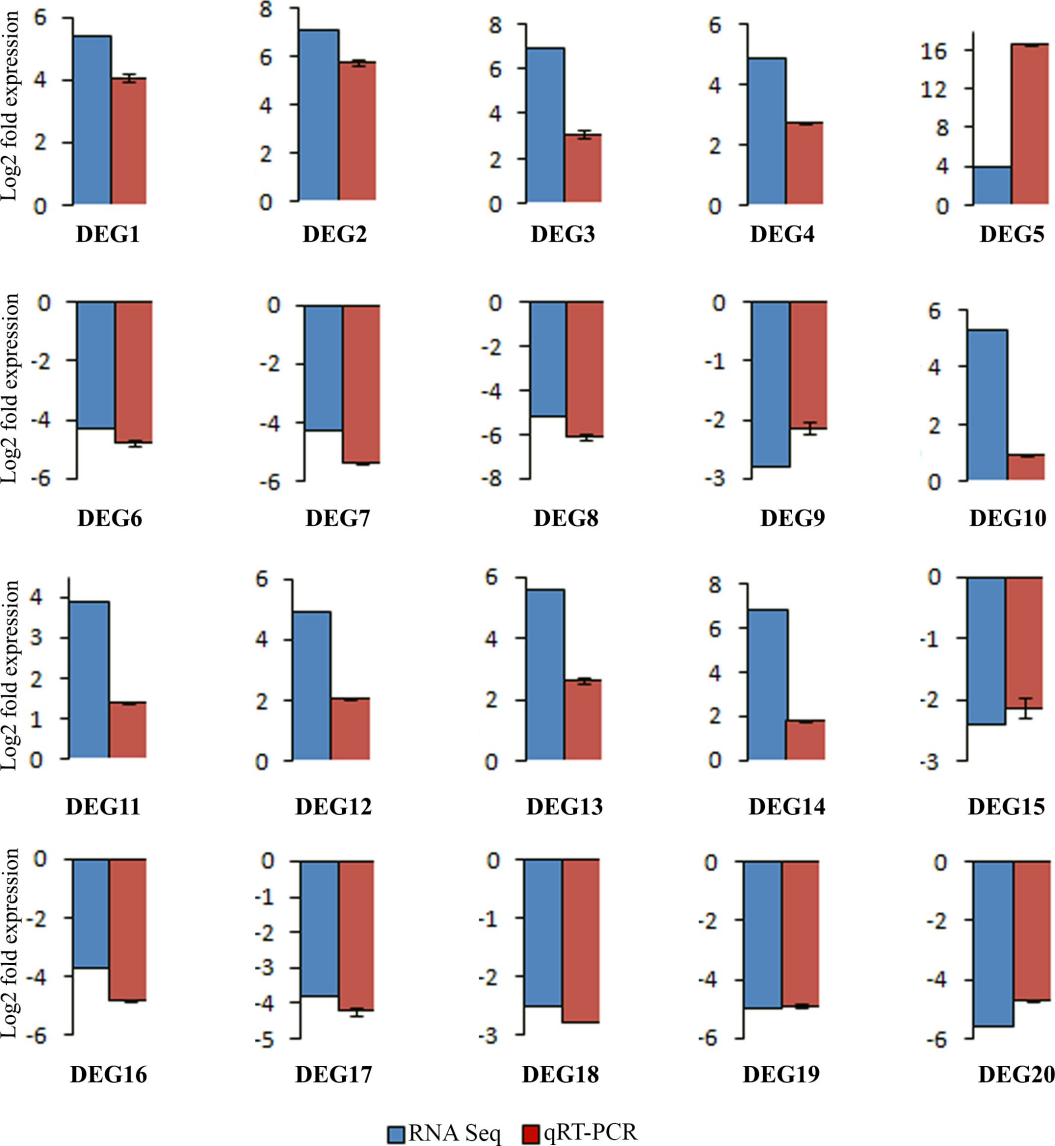
**Validation and comparison of the RNA-seq and qRT- PCR expression profile of differentially expressed genes** [Cysteine- rich receptor- like protein 3 (DEG1); G-type lectin receptor-like serine/threonine protein kinase (DEG2); Receptor-like protein kinase FERONIA (DEG3); Wall-associated receptor kinase-like 14 (DEG4); LysM domain receptor-like kinase 3 (DEG5); L-type lectin-domain containing receptor kinase VIII.2-like (DEG6); Transcription factor PIF3-like (DEG7); Heat stress transcription factor A-6b-like (DEG8); Protein LHY isoform X3 (DEG9); Transcription factor bHLH48-like (DEG10); calmodulin-binding transcription activator 1-like isoform X2 (DEG11); Probable WRKY transcription factor 41 (DEG12); probable methyltransferase 19 (DEG13); Subtilisin-like protease SBT1.6 (DEG14); U-box domain-containing protein 4 (DEG15); Zeatin expoxidase (DEG16); Delta-1-pyrroline-5-carboxylate synthase-like isoform X2 (DEG17); Flavonol synthase/flavanone 3-hydroxylase (DEG18); Probable inositol transporter 2 isoform X2 (DEG19); B-box zinc finger protein 18-like (DEG20)].

Twenty genes (Table A in S1 File) selected for qRT-PCR mainly belonged to transcription factors, receptor like kinases and genes involved in secondary metabolism as well as those that were identified through network analysis. These genes were selected based on their significance in differential expression between the two species as revealed by RNA-Seq analysis. Among receptor like kinases, Cysteine-rich receptor-like protein kinase, G-type lectin S-receptor-like serine/threonine-protein kinase, Receptor-like protein kinase FERONIA, wall-associated receptor kinase-like 14 and lysM domain receptor-like kinase 3 that were displaying up-regulation in the wild relative were chosen. Whereas, L-type lectin-domain containing receptor kinase VIII.2-like that showed down-regulation were also selected for validation. Transcription factor bHLH48-like, calmodulin-binding transcription activator 1-like isoform X2 and probable WRKY transcription factor 41 which showed up-regulation and transcription factor PIF3-like, heat stress transcription factor A-6b-like, B-box zinc finger protein 18-like and protein LHY isoform X3 which showed down-regulation in the wild relative were the genes chosen among transcription factors. Genes pertaining to protein modification were, probable methyltransferase PMT19 and Subtilisin-like protease showing up-regulation and U-box domain-containing protein which was down-regulated in the wild relative were chosen for qRT-PCR. The analysis demonstrated strong corroboration between the two expression analyses indicating authenticity of the RNA seq analysis (Fig 8).

## Discussion

Plant domestication has been a process where plants with altered morphological and physiological traits have evolved to meet human requirements like yield, harvest and edibility/palatability [22, 23]. With time and rigorous selection process, there has been a reduction in the level of genetic variation amongst the cultivated varieties of different crops that are economically important [6]. As a result, the wild moved apart from the cultivated species and got placed in secondary or tertiary gene pools based on their crossability with cultivated species [6, 45]. The advent and surge in various biotechnological tools, especially omics-based applications, have enabled proficient sequencing of crop wild relatives for their use in crop improvement [12, 29–31].

Among the wild relatives in the incompatible tertiary gene pool, *C*. *platycarpus* has received considerable attention because of many desirable traits important for pigeonpea improvement [14, 18, 46]. Though preliminary efforts have been made towards deciphering the molecular scenario in the wild relatives of pigeonpea, there is still deficiency in the in-depth understanding of molecular signatures leading to various traits of importance. A comparative transcriptomic profiling would therefore depict the specific molecular structure in each of the cases and provide leads for a better comprehension of major differences between them [26–28]. The present study is the very first of its kind and is an initiation towards not only generation of genomic resources in *C*. *platycarpus* but also a step towards exploitation of the identified resources in crop improvement programs. The choice of *C*. *platycarpus* accession, ICPW068 in the present study was because of its potential to resist various stresses [13, 14, 16, 19, 20] while TTB-7, a medium duration high yielding variety susceptible to various diseases and pests of pigeonpea including pod borer [24, 47, 48].

Expression profiling is better exploited in actively growing stages of plants. In pigeonpea, the epitome of vegetative growth happens during 45–60 days after sowing and at this stage of the crop growth, the response of plants to various stresses can be maximal [49, 50]. In the present study, leaf tissues from actively growing and healthy 45 days old TTB7 as well as *C*. *platycarpus* were harvested for transcriptome analysis. Leaf tissue was chosen in the study with a broader aim of understanding the relevance of the wild relative in mitigating insect herbivory and the fact that the larvae of *H*. *armigera* initially feed on leaves before reaching the pod [24, 25].

The quality of leaf transcriptomes of both the species was found to be reliable with respect to parameters like N50, number and length of transcripts. Based on KEGG analysis, *C*. *cajan* was seen to focus more on energy metabolism and pathways required for normal cellular machinery. However, *C*. *platycarpus* was seen to be proactive in signal perception and transduction processes as the annotated genes in all major terms were related to cell signaling. Interestingly, *C*. *platycarpus* annotated with one of the pathway GO terms, "T cell receptor signalling pathway", which is commonly annotated in mammals, concomitantly called Toll/interleukin-1 receptor (TIR) homology domain in plants, an intracellular domain common among the identified plant R-proteins [51]. Similar to mammalian and insect TIR genes [52], plant TIR genes are also known to play a major role in plant innate immunity involved in the activation of transcription factors through adopter proteins/protein kinases. The GO analysis reported in this study is the first of its kind where transcriptome of the cultivated pigeonpea was compared with its wild relative, though some studies have been carried out in other legumes [53–56].

In-depth analysis of the transcriptome would be definitely fascinating for a better perception of the species under study. Towards this, MapMan, an advanced bioinformatics tool for comprehensive interpretation of transcriptome data and visualization of functions of associated genes was used. This analysis allowed us to explore gene categories from the large data sets to get meaningful information. Through MapMan, it was evident that significant variation between the two species was conspicuous with respect to genes related to transcription factors (TFs), signaling, secondary metabolites, and stress response. Exploration of specific bins was attempted in order to decipher major similarities as well as differences between the two systems.

In general, TFs are seen to be involved in various plant processes like growth, development and stress signaling [26, 57–59]. Interestingly, MapMan analysis showed that the wild relative, *C*. *platycarpus* expressed more number of WRKY transcripts when compared to its cultivated counterpart depicting its role in regulation of various abiotic and biotic responses [60, 61]. Similarly, the wild relative also portrayed higher expression of another TF, MYB that plays an important role not only in plant development but also stress mitigation [62] indicating inherent agility of the wild relative.

Further support for this assumption was established based on the analysis of genes involved in cellular signal transduction. Response of plants to various environmental and developmental signals is pertinent for successful growth and reproduction [63, 64]. Proactive response to environmental/developmental cues was explicitly depicted in *C*. *platycarpus* as a large number of varied kinases, especially receptor like kinases and those involved in calcium-mediated signaling [65] were seen to be inherently up-regulated. Furthermore, it was also seen that the wild relative expressed a large number of transcripts coding for numeral G-proteins. Information accruing from literature implicates G-proteins with various functional processes including response to growth, development and environmental cues [66, 67].

Secondary metabolism produces a large number of specialized molecules that are required for the plant to survive in its environment and essential for communicating with other organisms in a mutualistic (eg. to attract beneficial organisms such as pollinators) or antagonistic (eg. to combat herbivores and pathogens) manner. Under baseline or non-stress conditions, it is expected that mutualistic metabolites or those required for normal physiological processes are expressed [68, 69]. This scenario was unambiguously seen in the present study as major differences were not observed between the two species. However, tocopheral biosynthesis genes were interestingly seen to be upregulated in *Cajanus platycarpus*. Tocopheral plays a crucial role in wax accumulation in plant leaves. It is a known fact about *C*. *platycarpus* that it portrays more pubescence, increased hardening of leaves (sclerophyly) by cuticular wax accumulation, cell wall thickening and lignifications. These traits are expected to prevent plants from insect attack by making them non-preferable, unpalatable and undigestable [16, 70, 71]. The increase accumulation of tocopherol can be extrapolated to the specific phenotype of the wild relative and it being a deterrent to insects.

Furthermore, another interesting feature observed was the variation in the transcripts pertaining to biotic and abiotic stress. Though, the study did not involve imposition of stress, still majority of stress-related gene transcripts were seen to be up-regulated in *C*. *platycarpus*. This variation presented in the study repeatedly depicted intrinsic differences between the two species at transcriptome level thus reconfirming earlier evidences in other categories like TFs and signaling.

Perfect corroboration was evident from interactions between the differentially expressed genes of specific bins derived from MapMan analysis. The inherent variation in the kind and specific function of transcripts between the two species was clear when it was observed that distinct clusters densely packed with transcripts dominated by *C*. *platycarpus* were found to be interacting in the developed network.

Therefore, considering different aspects of the study, clear disparity was seen in the transcriptome profiles of the two pigeonpea species, with the wild relative demonstrating skewed expression of transcripts pertaining to signaling, transcription factors and certain biotic stress related genes. However, dynamics of the transcriptome under specific stress conditions will provide intriguing insights and reasoning for the variety of desirable agronomic traits persisting in the wild relative. This learning can be a platform for further investigations with respect to the wild relative in deciphering the hidden molecular mechanisms towards mitigation of various biotic/abiotic stresses.

## Supporting information

S1 File **Table A.** List of primers used in the study; **Table B.** QC statistics; **Table C.** Assembly statistics.
(DOCX)

S1 Data **Table A.** Complete list of transcripts with annotation; **Table B.** Differential gene expression analysis of *C*. *platycarpus* vs *C*. *cajan* (given in separate tabs).
(XLSX)

S2 Data Species specific expression of transcripts under the selected MapMan bins. (XLSX)

S3 Data List of differentially expressed transcripts between *C. platycarpus* and C. cajan under selected MapMan bins. (XLSX)

S4 Data Summary of transcripts selected for network analysis. (XLSX)
