# Supplementary material for: Comparative transcriptome analysis of pigeonpea, Cajanus cajan (L.) and one of its wild relatives Cajanus platycarpus (Benth.) Maesen
Source: PLoS One. 2019 Jul 3;14(7):e0218731. doi: 10.1371/journal.pone.0218731 (PMC6609033; doi:10.1371/journal.pone.0218731)
Supplement: S1 File — Table A. List of primers used in the study; Table B. QC statistics; Table C. Assembly statistics. (DOCX) [file pone.0218731.s001.docx]

**Table A.** List of primers used in the study

| **Transcript ID** | **Primer ID** | **Gene name** | **Primer sequemce (5'-3')** | **Amblicon size (bp)** |
| --- | --- | --- | --- | --- |
| CP_TR31433\|c0_g1_i5_len=2589 | DEG 1 | Cysteine-rich receptor-like protein kinase | GTCTACCTTCACGAGGAATCAC | 125 |
|  |  |  | TCTGGGAACAATCTAGCAAGTC |  |
| CP_TR32556\|c0_g1_i1_len=2484 | DEG 2 | G-type lectin S-receptor-like serine/threonine-protein kinase | TAGAGATGGAAGCAACGACAAG | 102 |
|  |  |  | AGGTTCCACCCTAGTTTCATTC |  |
| CP_TR32659\|c1_g1_i4_len=3218 | DEG 3 | Receptor-like protein kinase FERONIA | ACTGAAAGGCCAGATAACACC | 97 |
|  |  |  | GTTCATGGAAGGTCGCTGAG |  |
| CP_TR22760\|c0_g2_i2_len=2065 | DEG 4 | wall-associated receptor kinase-like 14 | CAAGGAGACTCACCATTGCTAC | 128 |
|  |  |  | TGATTCACAGTTGTAGTCCAAGAG |  |
| CP_TR443\|c0_g2_i1_len=1474 | DEG 5 | lysM domain receptor-like kinase 3 | GTTCAGGTTCGACGAGAAGAC | 114 |
|  |  |  | AATCTCCGATCCACCCAATTC |  |
| CP_TR37415\|c0_g1_i1_len=1865 | DEG 6 | L-type lectin-domain containing receptor kinase VIII.2-like | CAGTGGAGTTTGACACAAGAAAG | 127 |
|  |  |  | TTAAGGGACACTCCAACATCAG |  |
| CP_TR37911\|c0_g1_i1_len=635 | DEG 7 | transcription factor PIF3-like | ATCCTCCCAAAGAAAGAACTCC | 133 |
|  |  |  | CTTGAGAGGAGATGAAGGTAAGC |  |
| CP_TR10174\|c0_g2_i1_len=1176 | DEG 8 | heat stress transcription factor A-6b-like | CCTGCCTTCATATACCAACTCC | 115 |
|  |  |  | TGCTTGATTCTCCAACACCTC |  |
| CP_TR29773\|c0_g1_i2_len=2457 | DEG 9 | protein LHY isoform X3 | GCACGTTGTTGATGGGAATAG | 117 |
|  |  |  | GCAGAAGTGGTGACAAGATTAG |  |
| CP_TR10148\|c0_g1_i1_len=1122 | DEG 10 | Transcription factor bHLH48-like | GGGCTAGGAGAGAGAAGATAAATG | 123 |
|  |  |  | GTAGAGATTGCACATGGTTGATG |  |
| CP_TR29370\|c0_g3_i1_len=3564 | DEG 11 | calmodulin-binding transcription activator 1-like isoform X2 | TATTTGATCGGAAGGTGCTGAG | 116 |
|  |  |  | ATCCACACTTCCAGCCTTTAG |  |
| CP_TR8901\|c0_g1_i1_len=1585 | DEG 12 | probable WRKY transcription factor 41 | TCTGTTCATGGAAGTCCTTTGAG | 105 |
|  |  |  | CATCCATTTGGGCATTGTCTTTC |  |
| CP_TR23836\|c0_g1_i2_len=1334 | DEG 13 | probable methyltransferase PMT19 | GACTGCTTATGATGGGCTTTAC | 115 |
|  |  |  | TTGCCACGCATTGTAGTTTAC |  |
| CP_TR54168\|c0_g1_i1_len=1854 | DEG 14 | Subtilisin-like protease | CTCCCGGTGTCAACATCCTC | 118 |
|  |  |  | TGAGGGCAAGCCATGGAAG |  |
| CP_TR17127\|c0_g1_i1_len=1128 | DEG 15 | U-box domain-containing protein 4 | CGGCATCATTCTTGAGACAAAC | 136 |
|  |  |  | GGCAGTTCTTCCCTCATCATAC |  |
| CP_TR29112\|c1_g1_i1_len=1718 | DEG 16 | zeaxanthin epoxidase | ATGGTCCAAGGTGAGGAATAAG | 117 |
|  |  |  | CGGTATCCAACAGTTTCAATGTC |  |
| CP_TR26975\|c0_g1_i1_len=2129 | DEG 17 | delta-1-pyrroline-5-carboxylate synthase-like isoform X2 | TCCTCCAAGTGACCCAAATTC | 123 |
|  |  |  | GCATTAACTTTGGCAGTCATACC |  |
| CP_TR60158\|c0_g1_i1_len=580 | DEG 18 | flavonol synthase/flavanone 3-hydroxylase | TGGTCACTGGTACGATGTTAAG | 129 |
|  |  |  | GTCTGCTCTTTGTTCACTGTTG |  |
| CP_TR8356\|c0_g1_i1_len=289 | DEG 19 | probable inositol transporter 2 isoform X2 | CCAATATGGATGGCTTGCATTG | 127 |
|  |  |  | TCCACATATCCCTCTGTACCTTAG |  |
| CP_TR22428\|c0_g1_i1_len=694 | DEG 20 | B-box zinc finger protein 18-like | CTGCTTTCTTCTATTGCGAGAC | 123 |
|  |  |  | GAAACTCAACTCTCTGCCTAAAC |  |

**Table B.** QC Statistics

| **Sample Names** | **Raw Reads** | **HQ Reads** | **% HQ Reads** | **(G+C)%** |
| --- | --- | --- | --- | --- |
| CC_0hr_C1a | 16771062 | 16187138 | 96.52% | 45.83% |
| CC_0hr_C1b | 17484123 | 16964438 | 97.03% | 45.66% |
| CP_0hr_P1a | 16227734 | 15777705 | 97.23% | 46.09% |
| CP_0hr_P1b | 16618450 | 16109862 | 96.94% | 45.98% |

**Table C.** Assembly statistics

| **Parameter** | ***Cajanus cajan* Assembly** | ***Cajanus platycarpus* Assembly** | **Combined Assembly after CD-Hit analysis** |
| --- | --- | --- | --- |
| Total No. of transcripts | 64214 | 64086 | 114781 |
| Total bases | 67919901 (~67.9 Mb) | 64684965 (~64.7 Mb) | 112954209 (~113 Mb) |
| Min transcript length | 224 | 224 | 224 |
| Max transcript length | 13591 | 13783 | 13783 |
| Average transcript length | 1057.71 | 1009.35 | 984.08 |
| N50 length | 1459 | 1433 | 1382 |
| (G + C)s % | 41.90% | 42.71% | 42.20% |
